# 1-Methyl-1*H*-2,1-benzothia­zin-4(3*H*)-one 2,2-dioxide

**DOI:** 10.1107/S1600536808003498

**Published:** 2008-02-06

**Authors:** M. Nawaz Tahir, Muhammad Shafiq, Islam Ullah Khan, Waseeq Ahmad Siddiqui, Muhammad Nadeem Arshad

**Affiliations:** a University of Sargodha, Department of Physics, Sargodha, Pakistan; b Government College University, Department of Chemistry, Lahore, Pakistan; c University of Sargodha, Department of Chemistry, Sargodha, Pakistan

## Abstract

In the crystal structure of the title compound, C_9_H_9_NO_3_S, there is distorted tetra­hedral geometry around the S atom. The sulfonyl group is almost normal to the benzene ring, while the carbonyl O atom and methyl C atom are on opposite sides of this ring. The heterocyclic ring adopts a half-boat conformation with the S atom out of the plane. The mol­ecules are dimerized by hydrogen bonding involving the benzene ring and the sulfonyl group. These dimers are linked to each other in the same way. There is an intra­molecular hydrogen bond between a methyl C—H group and a sulfonyl O atom, and a π–π inter­action between the aromatic rings of two dimers at a centroid-to-centroid distance of 3.6373 (13) Å.

## Related literature

For related literature, see: Cremer & Pople (1975 ▶); Harmata *et al.* (2004 ▶); Lombardino (1972 ▶); Misu & Togo (2003 ▶); Shafiq *et al.* (2008 ▶); Siddiqui *et al.* (2007 ▶).
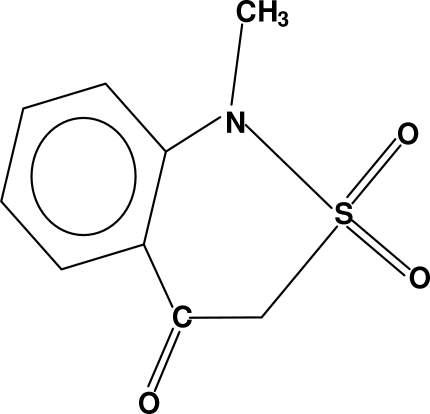


## Experimental

### 

#### Crystal data

C_9_H_9_NO_3_S
*M* *_r_* = 211.23
Triclinic, 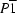

*a* = 7.4553 (4) Å
*b* = 8.5437 (4) Å
*c* = 8.7097 (4) Å
α = 67.691 (2)°
β = 70.467 (2)°
γ = 66.327 (2)°
*V* = 459.09 (4) Å^3^
*Z* = 2
Mo *K*α radiation
μ = 0.33 mm^−1^
*T* = 296 (2) K
0.20 × 0.15 × 0.10 mm

#### Data collection

Bruker Kappa APEXII CCD diffractometer
Absorption correction: multi-scan (*SADABS*; Bruker, 2007 ▶) *T*_min_ = 0.952, *T*_max_ = 0.971
7205 measured reflections
1787 independent reflections
1678 reflections with *I* > 2σ(*I*)
*R*_int_ = 0.019

#### Refinement

*R*[*F* ^2^ > 2σ(*F* ^2^)] = 0.032
*wR*(*F* ^2^) = 0.095
*S* = 1.09
1678 reflections
127 parameters
H-atom parameters constrained
Δρ_max_ = 0.22 e Å^−3^
Δρ_min_ = −0.42 e Å^−3^

### 

Data collection: *APEX2* (Bruker, 2007 ▶); cell refinement: *APEX2*; data reduction: *SAINT* (Bruker, 2007 ▶); program(s) used to solve structure: *SHELXS97* (Sheldrick, 2008 ▶); program(s) used to refine structure: *SHELXL97* (Sheldrick, 2008 ▶); molecular graphics: *ORTEP-3 for Windows* (Farrugia, 1997 ▶) and *PLATON* (Spek, 2003 ▶); software used to prepare material for publication: *WinGX* (Farrugia, 1999 ▶) and *PLATON*.

## Supplementary Material

Crystal structure: contains datablocks global, I. DOI: 10.1107/S1600536808003498/bq2063sup1.cif

Structure factors: contains datablocks I. DOI: 10.1107/S1600536808003498/bq2063Isup2.hkl

Additional supplementary materials:  crystallographic information; 3D view; checkCIF report

## Figures and Tables

**Table 1:** Hydrogen-bond geometry (Å, °)

*D*—H⋯*A*	*D*—H	H⋯*A*	*D*⋯*A*	*D*—H⋯*A*
C2—H2⋯O1^i^	0.93	2.58	3.432 (2)	152
C4—H4⋯O2^ii^	0.93	2.46	3.238 (3)	142
C9—H9*A*⋯O2	0.96	2.31	2.830 (3)	113

Symmetry codes: (i) 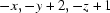; (ii) 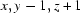.

**Fig. 1.**
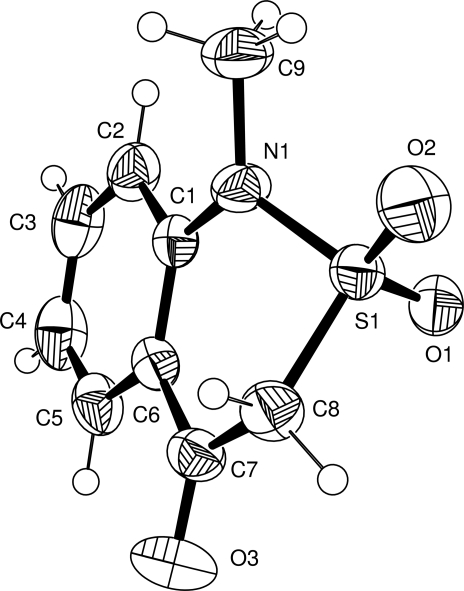
ORTEP drawing of the title compound, C10H11NO3S, with the atom numbering scheme. The thermal ellipsoids are drawn at the 50% probability level.

**Fig. 2.**
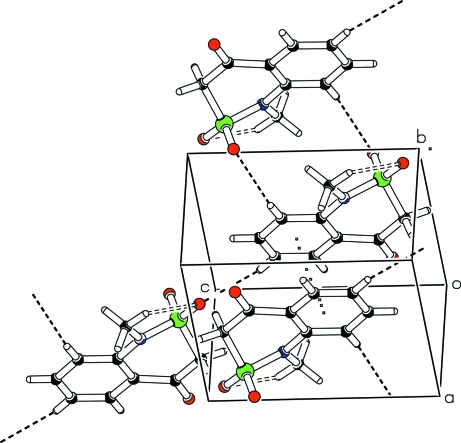
The packing figure (PLATON: Spek, 2003) which shows the dimeric nature of the compound owing to inter molecular hydrogen bondings and also showing a link between dimers.

## supplementary crystallographic information

### Comment

Benzothiazines belong to an important heterocyclic class of compounds which find a number of applications in medicinal chemistry. Derivatives of 2,1-benzothiazines were reported to possess potent biological activities such as lipoxygenase inhibition and are used as drugs for heart diseases (Misu, & Togo, 2003). These are used as intermediate precursors for the preparation of drugs used for curing Tuberculosis (Harmata *et al.*, 2004). The importance of 2,1-benzothiazine derivatives in medicinal chemistry has brought enormous attention to their synthesis. Herein, we report the crystal structure of the title compound.

The structure determination of the title compound (I) is in continuation to our research on derivatives of 2,1 benzothiazine (Shafiq *et al.*, 2008). The structural isomer (II), 2-methyl-2*H*-1,2-benzothiazin-4(3*H*)one 1,1-dioxide of (I) has been reported (Siddiqui *et al.*, 2007).

In the title compound the bond distances S1—N1 [1.6429 (13) Å] and S1—C8 [1.7514 (17) Å], have been significantly changed in comparison to 1.629 (3) Å and 1.760 (4) Å respectively as reported in (II). The change in bond angles around S is large enough which is evident from the range [99.47 (8)°-118.32 (8)°] in (I), as compared to (II) [103.05 (16)°- 118.66 (16)°]. All the atoms in heterocyclic thiazine as well as C-atoms of phenyl ring are nearly planer except that of S1. The S1 is displaced by 0.7834 (15) Å from the plane (*a*) defined by (C1 to C8, N1) and C-atom of methyl group is at a distance of 0.340 (3) Å from it. Thus the atoms of the two rings form a long half boat confirmation. The plane (*b*) defined by the atoms (O1,S1,O2) makes a dihedral angle of 89.81 (6)° to the plane (*a*) and hence these two planes are almost normal to each other. The O1-atom is at longest distance of -2.1912 (16) Å from plane (*a*). In the asymmetric unit there is an intramolecular H-bond as given in Table 2. The confirmation of the hetrocyclic ring in terms of the puckering parameters (Cremer & Pople, 1975) is described by; Q = 0.5767 (14) Å, θ = 54.98 (16)° and φ = 359.7 (2)°. The title compound is basically dimerized by H-bonding through C2—H2···O1^i^ (i = -*x*, -*y* + 2, -*z* + 1) as shown in Fig 2. It is interesting that the ring formed in dimer is of twelve bonds to which the methyl groups adopt *cis*, *trans* position. The dimers are linked to each other by symmetry code ii = *x*, *y* - 1, *z* + 1. The closest interaction [3.283 (2) Å] occurs between O1···O1^iii^ (symmetry code: iii = -*x*, -*y* + 2, -*z*), other than atoms involved in intermolecular H-bond. There is no *X*—H···*Cg* bond, however there exist a π-π interaction between the aromatic rings of two dimers at a distance of 3.6373 (13) Å by the symmetry operation 1 - *x*, 1 - *y*, 1 - *z*.

### Experimental

The title compound (I) was synthesized using the reported procedure (Lombardino, 1972) and crystals were grown by slow evaporation from a solution of CH_3_OH at 298 K.

### Crystal data

**Table:**

C_9_H_9_NO_3_S	*Z* = 2
*M**_r_* = 211.23	*F*_000_ = 220
Triclinic, *P*1	*D*_x_ = 1.528 Mg m^−^^3^
Hall symbol: -P 1	Mo *K*α radiation radiation λ = 0.71073 Å
*a* = 7.4553 (4) Å	Cell parameters from 1678 reflections
*b* = 8.5437 (4) Å	θ = 2.6–25.9º
*c* = 8.7097 (4) Å	µ = 0.33 mm^−^^1^
α = 67.691 (2)º	*T* = 296 (2) K
β = 70.467 (2)º	Prismatic, colourless
γ = 66.327 (2)º	0.20 × 0.15 × 0.10 mm
*V* = 459.09 (4) Å^3^	

### Data collection

**Table:**

Bruker Kappa-APEXII CCD diffractometer	1787 independent reflections
Radiation source: fine-focus sealed tube	1678 reflections with *I* > 2σ(*I*)
Monochromator: graphite	*R*_int_ = 0.020
Detector resolution: 7.50 pixels mm^-1^	θ_max_ = 25.9º
*T* = 296(2) K	θ_min_ = 2.6º
ω scans	*h* = −9→9
Absorption correction: multi-scan(SADABS; Bruker, 2007)	*k* = −10→10
*T*_min_ = 0.952, *T*_max_ = 0.971	*l* = −10→10
7205 measured reflections	

### Refinement

**Table:**

Refinement on *F*^2^	Secondary atom site location: difference Fourier map
Least-squares matrix: full	Hydrogen site location: inferred from neighbouring sites
*R*[*F*^2^ > 2σ(*F*^2^)] = 0.032	H-atom parameters constrained
*wR*(*F*^2^) = 0.095	*w* = 1/[σ^2^(*F*_o_^2^) + (0.0531*P*)^2^ + 0.1411*P*] where *P* = (*F*_o_^2^ + 2*F*_c_^2^)/3
*S* = 1.09	(Δ/σ)_max_ < 0.001
1678 reflections	Δρ_max_ = 0.22 e Å^−^^3^
127 parameters	Δρ_min_ = −0.42 e Å^−^^3^
Primary atom site location: structure-invariant direct methods	Extinction correction: none

### Special details

**Table:**

Geometry. All e.s.d.'s (except the e.s.d. in the dihedral angle between two l.s. planes) are estimated using the full covariance matrix. The cell e.s.d.'s are taken into account individually in the estimation of e.s.d.'s in distances, angles and torsion angles; correlations between e.s.d.'s in cell parameters are only used when they are defined by crystal symmetry. An approximate (isotropic) treatment of cell e.s.d.'s is used for estimating e.s.d.'s involving l.s. planes.
Refinement. Refinement of *F*^2^ against ALL reflections. The weighted *R*-factor *wR* and goodness of fit *S* are based on *F*^2^, conventional *R*-factors *R* are based on *F*, with *F* set to zero for negative *F*^2^. The threshold expression of *F*^2^ > σ(*F*^2^) is used only for calculating *R*-factors(gt) *etc*. and is not relevant to the choice of reflections for refinement. *R*-factors based on *F*^2^ are statistically about twice as large as those based on *F*, and *R*- factors based on ALL data will be even larger.

### Fractional atomic coordinates and isotropic or equivalent isotropic displacement parameters (Å^2^)

**Table:**

	*x*	*y*	*z*	*U*_iso_*/*U*_eq_	
S1	0.18773 (6)	0.94371 (5)	0.15987 (5)	0.03811 (16)	
O1	−0.02000 (18)	0.95817 (16)	0.20416 (15)	0.0472 (3)	
O2	0.2454 (2)	1.09673 (18)	0.05037 (17)	0.0618 (4)	
O3	0.3153 (2)	0.46358 (19)	0.16958 (19)	0.0641 (4)	
N1	0.2759 (2)	0.87608 (18)	0.33158 (17)	0.0410 (3)	
C1	0.2522 (2)	0.71412 (19)	0.45108 (18)	0.0327 (3)	
C2	0.2208 (3)	0.6907 (2)	0.6241 (2)	0.0447 (4)	
H2	0.2151	0.7814	0.6616	0.054*	
C3	0.1982 (3)	0.5318 (3)	0.7405 (2)	0.0540 (5)	
H3	0.1782	0.5166	0.8559	0.065*	
C4	0.2048 (3)	0.3964 (3)	0.6881 (2)	0.0548 (5)	
H4	0.1883	0.2908	0.7672	0.066*	
C5	0.2359 (2)	0.4185 (2)	0.5186 (2)	0.0462 (4)	
H5	0.2398	0.3270	0.4833	0.055*	
C6	0.2618 (2)	0.57543 (19)	0.39688 (19)	0.0341 (3)	
C7	0.2997 (2)	0.5863 (2)	0.2155 (2)	0.0399 (4)	
C8	0.3241 (3)	0.7571 (2)	0.0823 (2)	0.0434 (4)	
H8A	0.2785	0.7728	−0.0162	0.052*	
H8B	0.4650	0.7481	0.0465	0.052*	
C9	0.3194 (3)	1.0018 (3)	0.3773 (3)	0.0555 (5)	
H9A	0.3301	1.1026	0.2801	0.083*	
H9B	0.4436	0.9450	0.4149	0.083*	
H9C	0.2133	1.0409	0.4671	0.083*	

### Atomic displacement parameters (Å^2^)

**Table:**

	*U*^11^	*U*^22^	*U*^33^	*U*^12^	*U*^13^	*U*^23^
S1	0.0511 (3)	0.0305 (2)	0.0313 (2)	−0.01201 (17)	−0.01384 (17)	−0.00395 (16)
O1	0.0486 (7)	0.0447 (7)	0.0460 (7)	−0.0040 (5)	−0.0179 (5)	−0.0152 (5)
O2	0.0921 (11)	0.0419 (7)	0.0480 (7)	−0.0301 (7)	−0.0247 (7)	0.0087 (6)
O3	0.0813 (10)	0.0563 (8)	0.0664 (9)	−0.0287 (7)	0.0007 (7)	−0.0364 (7)
N1	0.0614 (9)	0.0327 (7)	0.0371 (7)	−0.0211 (6)	−0.0205 (6)	−0.0033 (6)
C1	0.0317 (7)	0.0311 (7)	0.0322 (7)	−0.0082 (6)	−0.0092 (6)	−0.0055 (6)
C2	0.0462 (9)	0.0493 (10)	0.0356 (8)	−0.0100 (7)	−0.0121 (7)	−0.0113 (7)
C3	0.0454 (10)	0.0683 (13)	0.0313 (8)	−0.0154 (9)	−0.0091 (7)	0.0017 (8)
C4	0.0474 (10)	0.0477 (10)	0.0511 (11)	−0.0196 (8)	−0.0126 (8)	0.0116 (8)
C5	0.0414 (9)	0.0330 (8)	0.0580 (11)	−0.0132 (7)	−0.0129 (8)	−0.0029 (7)
C6	0.0304 (7)	0.0293 (7)	0.0386 (8)	−0.0084 (6)	−0.0068 (6)	−0.0072 (6)
C7	0.0364 (8)	0.0391 (8)	0.0458 (9)	−0.0101 (6)	−0.0046 (6)	−0.0193 (7)
C8	0.0500 (9)	0.0451 (9)	0.0317 (8)	−0.0113 (7)	−0.0055 (7)	−0.0141 (7)
C9	0.0770 (13)	0.0443 (10)	0.0624 (11)	−0.0274 (9)	−0.0267 (10)	−0.0143 (8)

### Geometric parameters (Å, °)

**Table:**

S1—O2	1.4262 (13)	C3—H3	0.9300
S1—O1	1.4302 (13)	C4—C5	1.365 (3)
S1—N1	1.6429 (13)	C4—H4	0.9300
S1—C8	1.7574 (17)	C5—C6	1.397 (2)
O3—C7	1.209 (2)	C5—H5	0.9300
N1—C1	1.4175 (18)	C6—C7	1.482 (2)
N1—C9	1.452 (2)	C7—C8	1.515 (2)
C1—C2	1.393 (2)	C8—H8A	0.9700
C1—C6	1.402 (2)	C8—H8B	0.9700
C2—C3	1.387 (2)	C9—H9A	0.9600
C2—H2	0.9300	C9—H9B	0.9600
C3—C4	1.375 (3)	C9—H9C	0.9600
			
O2—S1—O1	118.32 (8)	C4—C5—C6	121.56 (17)
O2—S1—N1	107.31 (8)	C4—C5—H5	119.2
O1—S1—N1	110.55 (7)	C6—C5—H5	119.2
O2—S1—C8	111.61 (9)	C5—C6—C1	118.92 (15)
O1—S1—C8	107.95 (8)	C5—C6—C7	117.93 (14)
N1—S1—C8	99.47 (8)	C1—C6—C7	123.14 (14)
C1—N1—C9	121.70 (14)	O3—C7—C6	122.71 (16)
C1—N1—S1	116.58 (10)	O3—C7—C8	118.80 (15)
C9—N1—S1	119.32 (12)	C6—C7—C8	118.48 (13)
C2—C1—C6	119.27 (14)	C7—C8—S1	111.72 (11)
C2—C1—N1	120.09 (14)	C7—C8—H8A	109.3
C6—C1—N1	120.63 (13)	S1—C8—H8A	109.3
C3—C2—C1	119.87 (17)	C7—C8—H8B	109.3
C3—C2—H2	120.1	S1—C8—H8B	109.3
C1—C2—H2	120.1	H8A—C8—H8B	107.9
C4—C3—C2	121.05 (17)	N1—C9—H9A	109.5
C4—C3—H3	119.5	N1—C9—H9B	109.5
C2—C3—H3	119.5	H9A—C9—H9B	109.5
C5—C4—C3	119.31 (16)	N1—C9—H9C	109.5
C5—C4—H4	120.3	H9A—C9—H9C	109.5
C3—C4—H4	120.3	H9B—C9—H9C	109.5
			
O2—S1—N1—C1	−174.04 (12)	C4—C5—C6—C1	−1.1 (2)
O1—S1—N1—C1	55.60 (13)	C4—C5—C6—C7	178.55 (15)
C8—S1—N1—C1	−57.74 (13)	C2—C1—C6—C5	1.2 (2)
O2—S1—N1—C9	23.23 (18)	N1—C1—C6—C5	−179.41 (14)
O1—S1—N1—C9	−107.13 (15)	C2—C1—C6—C7	−178.43 (14)
C8—S1—N1—C9	139.53 (15)	N1—C1—C6—C7	0.9 (2)
C9—N1—C1—C2	15.9 (2)	C5—C6—C7—O3	−2.5 (2)
S1—N1—C1—C2	−146.39 (13)	C1—C6—C7—O3	177.15 (16)
C9—N1—C1—C6	−163.48 (16)	C5—C6—C7—C8	178.83 (14)
S1—N1—C1—C6	34.24 (18)	C1—C6—C7—C8	−1.5 (2)
C6—C1—C2—C3	−0.5 (2)	O3—C7—C8—S1	151.98 (15)
N1—C1—C2—C3	−179.84 (14)	C6—C7—C8—S1	−29.30 (18)
C1—C2—C3—C4	−0.4 (3)	O2—S1—C8—C7	166.75 (12)
C2—C3—C4—C5	0.6 (3)	O1—S1—C8—C7	−61.58 (13)
C3—C4—C5—C6	0.2 (3)	N1—S1—C8—C7	53.77 (13)

### Hydrogen-bond geometry (Å, °)

**Table:**

*D*—H···*A*	*D*—H	H···*A*	*D*···*A*	*D*—H···*A*
C2—H2···O1^i^	0.93	2.58	3.432 (2)	152
C4—H4···O2^ii^	0.93	2.46	3.238 (3)	142
C9—H9A···O2	0.96	2.31	2.830 (3)	113

Symmetry codes: (i) −*x*, −*y*+2, −*z*+1; (ii) *x*, *y*−1, *z*+1.
